# Resistance or power training to enhance lower limb muscle morphology in ambulatory children with cerebral palsy? A focused systematic review with meta-analysis

**DOI:** 10.3389/fped.2025.1546156

**Published:** 2025-06-24

**Authors:** Bo Liu, Jizhi You, Yunxiang Fan, Yunping Xia, Xiang Zhang, Yang Zhang

**Affiliations:** ^1^ College of Physical Education, Hunan Normal University, Changsha, China; ^2^ College of Optical and Electronic Technology, China Jiliang University, Hangzhou, China; ^3^ Independent Researcher, Windermere, FL, United States

**Keywords:** exercise modalities, fascicle length, muscle hypertrophy, muscle mass, muscle volume, plyometric training, strength training

## Abstract

**Background:**

Early exercise interventions targeting lower limb muscles are critical for enhancing motor development in children with cerebral palsy (CP). While both resistance training, which enhances muscular strength and endurance, and power training, which targets explosive force production and movement velocity, fall under the umbrella of strength training, this focused review synthesizes current evidence on muscle hypertrophy resulting from these two modalities in children with CP.

**Methods:**

The Web of Science Core Collection, Scopus, and Embase were searched through March 2025. Eligible studies were randomized controlled trials assessing muscle fascicle length or proxy indicators of muscle fiber diameter following resistance or power training in children with CP. A random-effects meta-analysis was performed to calculate Cohen's *d* comparing strength training with regular physiotherapy.

**Findings:**

Eight studies met the inclusion criteria and were systematically reviewed, with five included in the meta-analysis. These five studies reported outcomes from 80 participants in the strength training group and 73 participants in the traditional physiotherapy group. All participants were ambulatory children classified with low to mild levels on the Gross Motor Function Classification System. Resistance training significantly increased muscle fiber diameter (*d* = 0.82, 95% CI = 0.54–1.09), whereas power training did not (*d* = 0.35, 95% CI = −0.29 to 0.99). Neither training modality produced a significant increase in muscle fascicle length (resistance training: *d* = 0.19, 95% CI = −0.17 to 0.56; power training: *d* = 0.37, 95% CI = −0.27 to 1.01). Notably, one study comparing power and resistance training demonstrated a highly significant improvement in muscle fascicle length (*d* = 1.20, 95% CI = 0.13–2.27), which may be attributed to the high-velocity, high-load nature of concentric power training.

**Interpretation:**

Current evidence favors resistance training to increase muscle fiber diameter in ambulatory children with CP. As individuals progress, maximal loads and repetitions should be progressively increased and complemented with explosive power training to further enhance muscle fascicle length and lower limb function. The optimal protocol for children with high levels of functional disability remains to be established.

## 1 Introduction

Cerebral palsy (CP) is a neurological disorder resulting from non-progressive brain damage or developmental defects during fetal or infant brain development, affecting approximately 1.5–3 per 1,000 live births globally (1). Preterm infants are at increased risk for cerebral white matter injury due to genetic factors, nutritional deficiencies, infections, or placental damage (2). These factors can trigger hypoxic-ischemic events, leading to CP via injuries to the cortex, subcortical regions, or basal ganglia. Additionally, birth-related ischemia, hypoxia, postnatal encephalitis, and cerebral hemorrhage may contribute to CP development. Clinically, CP is classified by manifestations such as spasticity, dyskinesia, ataxia, and abnormal tone, with spastic CP being the most common in children. Damage to neuromotor components disrupts skeletal muscle function (3), resulting in central motor deficits and postural abnormalities. Children aged 2–5 years with spastic CP exhibit approximately 22% lower muscle volume and markedly reduced lower-limb muscle cross-sectional area compared to their typically developing peers (4). Likewise, shortening of the rectus femoris—one of several affected muscles—reduces strength, contraction velocity, and range of motion, thereby impairing daily activities (5). Furthermore, skeletal muscles in CP frequently stiffen during activity, leading to weakness, imbalance, and reduced endurance, which hinders motor control and increases the risk of deformities. Ultimately, musculoskeletal malformations and muscle contractures significantly reduce quality of life, with persistent spasms often resulting in lifelong motor disabilities.

The absence of fixed deformities and high behavioral plasticity in young children with CP enables timely interventions to support muscular and motor development. Current treatments primarily involve long-term rehabilitation, such as gait training (6). Among traditional and emerging approaches, resistance training has gained interest as a non-invasive and cost-effective method to counter disease-related declines in muscle mass and function (7). Since weak lower limb muscles contribute to impaired gait, resistance training holds potential for improvement. Two systematic reviews support its benefits in enhancing strength, balance, and mobility in children with CP (8, 9). However, few studies have examined the impact of strength training on muscle morphology, with only one systematic review available that addresses overall strength training (10).

An important nuance in modern strength training lies in pairing exercise type with load and velocity to target strength-endurance, strength-speed, or speed-strength (11). Traditional resistance programs begin with a strength-endurance phase—refining technique and fortifying connective tissues to prepare novices for heavier loads—then progress to a strength-speed phase that maximizes force production and muscle hypertrophy via high loads. Advanced protocols add a speed-strength (power training) phase, using moderate loads with high-velocity exercises to maximize acceleration, movement velocity, and power output. In able-bodied trained children, power training yields superior gains in muscle mass (12), strength, power (13), and mobility (14) compared to traditional high-load resistance training, leading some researchers to advocate it as a replacement—rather than a complement—to resistance training for high-performance athletics (14). Restricted fiber-diameter growth in children with CP impairs both strength development and muscle power (7). This raises the question of whether power training—which involves rapid eccentric or concentric movements—offers advantages for muscle hypertrophy and function compared to resistance training. However, evidence of its effects in children with CP remains limited. van Vulpen et al. (15) examined power training targeting the plantar flexors using functionally loaded, multi-joint movements that emphasized ankle push-off, high movement velocity to simulate daily activities, and progressive loading at 50%–70% of maximum unloaded speed. Their assessments of isometric and dynamic muscle functions, along with muscle tone, provided preliminary evidence that moderate power output can enhance real-life functional capacities in children with CP. It should be kept in mind that, disuse-related muscle limitations in children with CP may restrict their ability to perform high-load—typically >80% of one-repetition maximum (1RM)—resistance training, while functional limitations may also impede rapid force exertion required for power training. Consequently, comparing these two strength training modalities is of significant interest. To our knowledge, no dedicated meta-analysis has yet addressed this specific micro aspect.

Therefore, the purpose of this systematic review was to summarize current evidence on the effects of resistance and power training on lower limb muscle morphology in children with CP. The findings are expected to foster academic dialogue and guide the optimization of muscle hypertrophy through evidence-based strength training protocols in clinical practice.

## 2 Methods

### 2.1 Search strategy

Two researchers (B.L. and Y.Z.) conducted an electronic search of the Web of Science Core Collection, Scopus, and Embase from inception to March 2025. The search combined the keywords “children” and “cerebral palsy” with either “exercise,” “training,” or “rehabilitation,” and further paired these with “muscle fiber diameter,” “muscle volume,” “muscle thickness,” “muscle size,” “cross-sectional area,” “muscle fiber length,” or “muscle fascicle length.”

Inclusion criteria comprised randomized controlled trials of resistance or power training interventions targeting lower-limb muscle morphology in CP participants aged 6–17 years (ambulatory or non-ambulatory) who had undergone no surgery in the preceding 12 months. Only full-length, English-language research articles were considered, while reviews, conference papers, and protocols were excluded. Two researchers (B.L. and X.Z.) independently screened the records and resolved disagreements by consensus.

### 2.2 Data extraction

Two researchers (B.L. and Y.Z.) extracted data using a standard sheet available on Figshare (doi: 10.6084/m9.figshare.28642094.v1). Extracted data included participant demographics, exercise characteristics, and outcomes of interest. Additionally, risk-of-bias information was recorded on a separate datasheet using ROB 2 tool (16).

Outcome measures included muscle fascicle length and proxy indicators of muscle fiber diameter—specifically muscle volume, thickness, or cross-sectional area. Typically, either the means with standard deviations or the mean change with paired *p*-values were extracted. Data reported as medians and interquartile ranges were converted to means and standard deviations using McGrath et al.'s quantile estimation method (metamedian, version 1.2.1) (17).

### 2.3 Data synthesis

Data were analyzed with the R package meta (version 8.0-2). Owing to heterogeneity among participants and interventions, we applied a random-effects meta-analysis and synthesized pooled effects as Cohen's d (18), despite the small sample sizes of the constituent studies. An effect was considered significant when its 95% confidence interval (CI) excluded zero. Heterogeneity was assessed using the *I*^2^ statistic. No subgroup analysis was conducted due to the inclusion of only one power training study. When a study reported multiple muscle morphology metrics, each outcome was treated as independent. To mitigate potential effect size inflation from multiple measurements within a single study, a sensitivity analysis using an inverse-variance weighted common effect size was conducted. Contour-enhanced funnel plots were used to visualize potential publication bias. Of note, two systematically reviewed studies were excluded from the meta-analysis because of control-arm inconsistencies (see “Results” for details); nonetheless, their control-adjusted effect sizes are discussed with the principal findings.

## 3 Results

**Figure 1:**
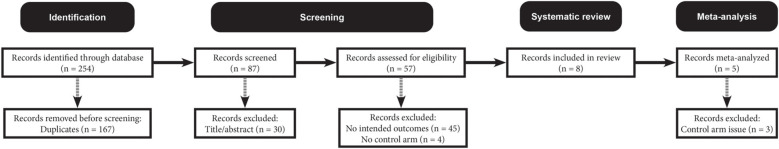
Flowchart of the literature search.

**Figure 2:**
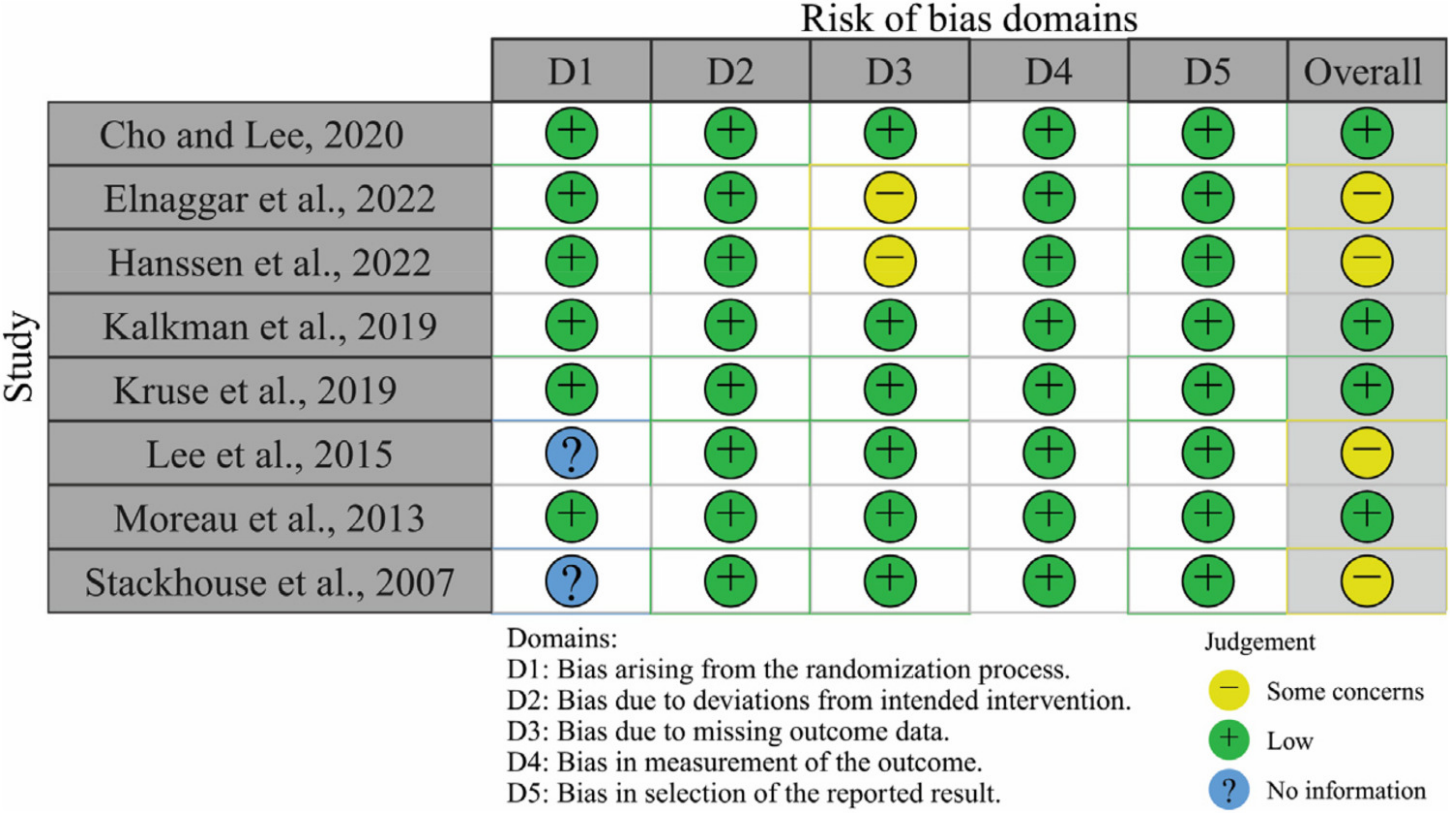
Risk of bias of all reviewed studies.

Figure 1 outlines the literature screening process. The search initially identified 254 unique records, which were subsequently narrowed down to eight eligible studies (19–26). Figure 2 visualizes the risk-of-bias assessment. Lee et al. (24) and Stackhouse et al. (26) did not specify their randomization methods, although this omission may not critically affect result validity. Elnaggar et al. (20) and Hanssen et al. (21) used partial imputation for missing data without detailing the affected data, raising concerns about outcome precision. Consequently, these studies present some issues in data presentation. Overall, the eight included studies qualify as valid randomized controlled trials, justifying their inclusion in the systematic review and meta-analysis.

Table 1 presents the training characteristics of all reviewed studies. Kruse et al. (23) and Moreau et al. (25) featured a control arm categorized as power training, and Stackhouse et al. (26) employed a typical resistance training control, while the remaining studies used regular physiotherapy as control. Although a network meta-analysis would be ideal, the limited evidence warranted a traditional meta-analysis comparing resistance or power training to regular physiotherapy. Consequently, five (19–22, 24) of the eight studies were included in the meta-analysis, with only Elnaggar et al. (20) categorized as power training.

**Table 1:** Training characteristics of systematically reviewed studies.

Source	Investigation arm	Control arm
Cho and Lee (19)	Progressive functional resistance training—increasing repetitions and load from 5%–35% of body weight—includes sit-to-stand, half-kneeling, and side-step-up exercises. Sessions are 30 min long, three times per week for 6 weeks	Functional exercises using a standing frame and mat routines are performed in 30-min sessions, three times per week for 6 weeks
Elnaggar et al. (20)	Plyometric power exercises include bounding, forward jumping, single-leg forward hopping, lateral leaping, side-to-side jumping, reciprocal stride jumping, squat jumping, tuck jumping, and high-step hopping. Sessions last 45 min, twice per week for 12 weeks	Physical therapy incorporates flexibility, strength, postural, and balance exercises, along with gait training. Sessions last 45 min, twice per week for 12 weeks
Hanssen et al. (21)	Progressive resistance training using resistance bands and weighted vests includes functional joint and muscle exercises. The protocol starts with 3 sets of 10 repetitions at approximately 60%–90% of 1RM, performed 3–4 times per week for 12 weeks	Usual care routine
Kalkman et al. (22)	Progressive resistance training begins with 4 weeks of standing heel raises performed at 3 sets of 12 repetitions, with a progressive increase in load via a weighted rucksack. This phase is followed by 6 weeks combining resistance training with calf stretching exercises. All sessions are conducted four times per week	Calf stretching exercises
Kruse et al. (23)	Progressive resistance training consists of functional lower limb exercises performed in circuits. Each circuit includes 10–12 repetitions of sit-to-stand, heel raises, forward lunges, lateral step-ups, and bridging exercises. Subsequently, an additional set of 10 repetitions is performed using a weighted vest. Training occurs three times per week for 8 weeks	The protocol is similar to the investigation arm except that high-intensity circuit training is used for resistance training. Exercise load was defined by the number of repetitions completed within a 30-s interval, with children instructed to perform as many repetitions as possible
Lee et al. (24)	In addition to a 30-min neurodevelopmental treatment, participants also received a 30-min resistance training session three times a week for 6 weeks. The functional program included sit-to-stand exercises—with an initial load of 5% of body weight that was progressively increased—as well as both loaded and unloaded lateral step-ups and half knee-rises	Participants underwent 30-min neurodevelopmental treatment sessions, three times per week for 6 weeks
Moreau et al. (25)	Power training consisted of six sets of five concentric exertions at 80% of maximum concentric force, performed at 30°/s during week 1, with velocity progressively increased to 120°/s. Sessions were conducted three times per week for a total of 24 sessions	The resistance training consisted of 3–5 submaximal efforts, followed by 6 sets of 5 maximal-effort concentric knee extension contractions at a velocity of 30°/s. Sessions were conducted three times per week for a total of 24 sessions
Stackhouse et al. (26)	Resistance training was performed using neuroelectrically elicited contractions without voluntary effort. Each isometric contraction lasted 15 s, followed by 45 s of rest. A total of 15 min of exercise was conducted for the quadriceps and triceps surae muscles, three times per week for 12 weeks	The protocol was similar to the investigation arm, except that no neuromuscular stimulation was applied, and exercise was performed using maximal voluntary isometric contractions

Table 2 summarizes the participant demographics of meta-analyzed studies. The intervention arm included 80 participants (51.3% boys), while the control arm comprised 73 participants (64.4% boys). All but one study recruited children with diparesic CP. Notably, all studies enrolled ambulatory children with lower gross motor function classification system (GMFCS) levels (I–III), indicating relatively adequate baseline mobility for participating in exercise training.

**Table 2:** Participant characteristics of meta-analyzed studies.

Source	Investigation arm				Control arm			
	N (b/g)	Age (y)	CP	GMFCS^a^	N (b/g)	Age (y)	CP	GMFCS^a^
Cho and Lee (19)	4/9	5.5	DP	70.0	8/4	7.2	DP	68.2
Elnaggar et al. (20)	11/8	13.0	DP	I × 13; II × 6	13/6	13.5	DP	I × 16; II × 3
Hanssen et al. (21)	14/12	8.3	DP	I × 17; II × 5; III × 4	16/6	8.5	DP	I × 14; II × 5; III × 3
Kalkman et al. (22)	4/5	11.1	DP/HP	I × 5; II × 4	5/2	8.5	DP/HP	I × 3; II × 3; III × 1
Lee et al. (24)	8/5	6.1	DP	78.0	5/8	6.9	DP	79.1

b/g, boy/girl; CP, cerebral palsy; DP, diparesis; GMFCS, gross motor function classification system; HP, hemiparesis.

^a^ Data are presented as mean scores (assessed by GMFCS-88) or as counts by level (I, II, III; n).

**Figure 3:**
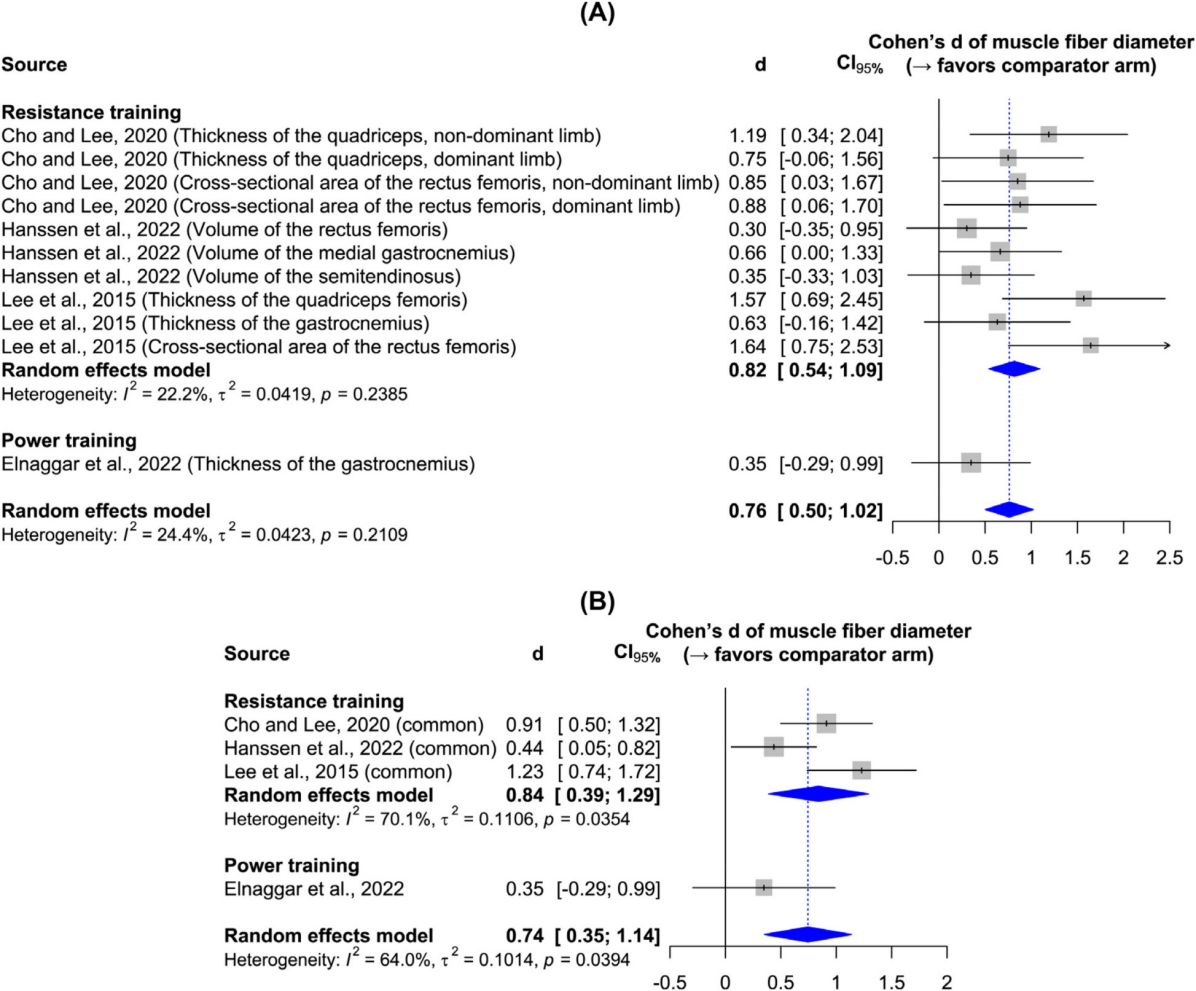
Forest plots: **(A)** main results of the effect on muscle fiber diameter; **(B)** sensitivity analysis.

**Figure 4:**
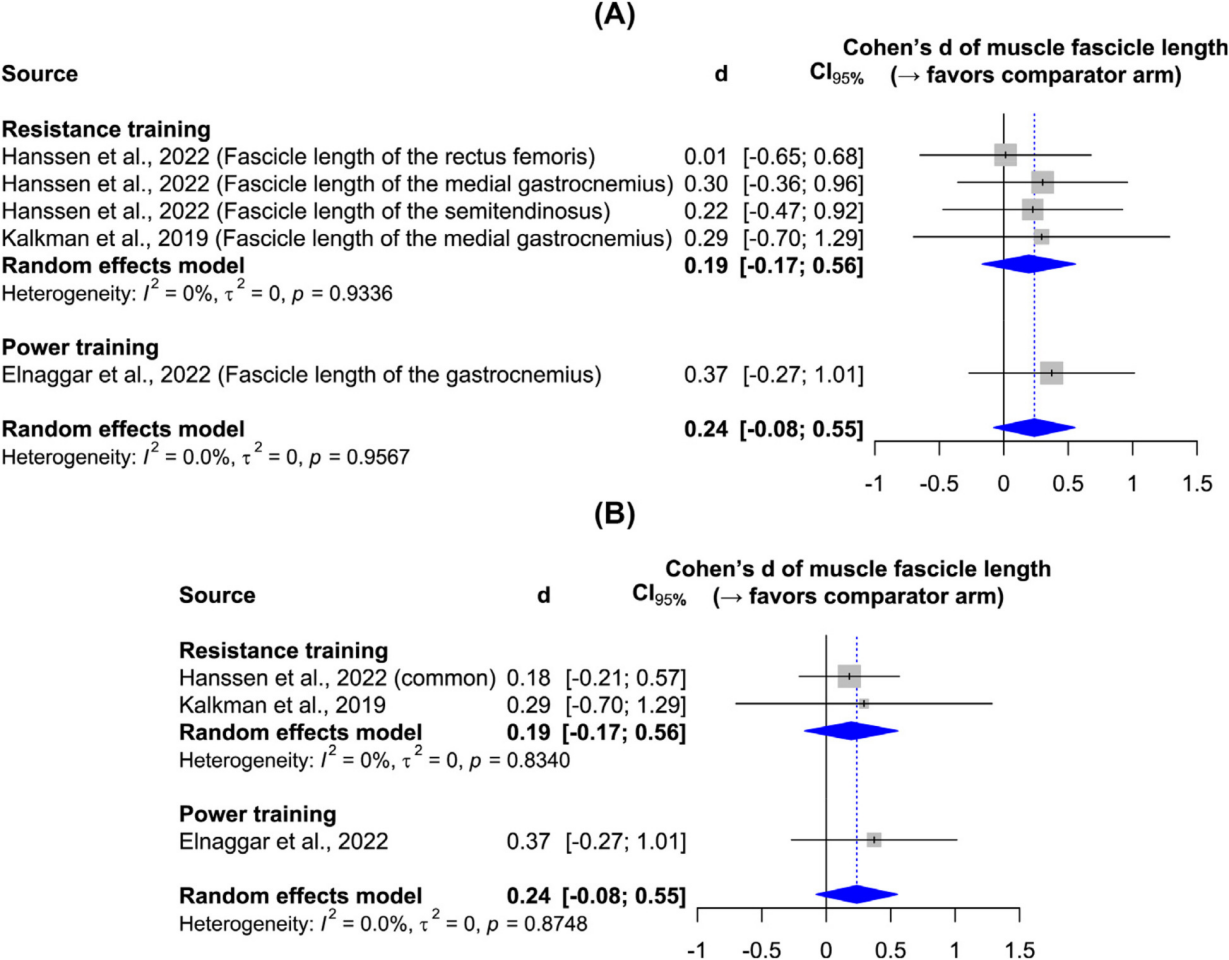
Forest plots: **(A)** main results of the effect on muscle fascicle length; **(B)** sensitivity analysis.

Figure 3 shows the effects of resistance and power training on muscle fiber diameter, as measured by ultrasonography in all studies. Resistance training yielded a significant large effect size, while the single power training study leaves it unclear whether plyometric exercises effectively increase muscle thickness. Sensitivity analysis confirms the robustness of the results to the data synthesis method. Although the main results indicate low heterogeneity, the sensitivity analysis reveals heterogeneity—an expected outcome given participant and intervention variations. Figure 4 shows the effect of resistance and power training on muscle fascicle length. Neither type of training yielded significant improvement over regular physiotherapy.

**Figure 5:**
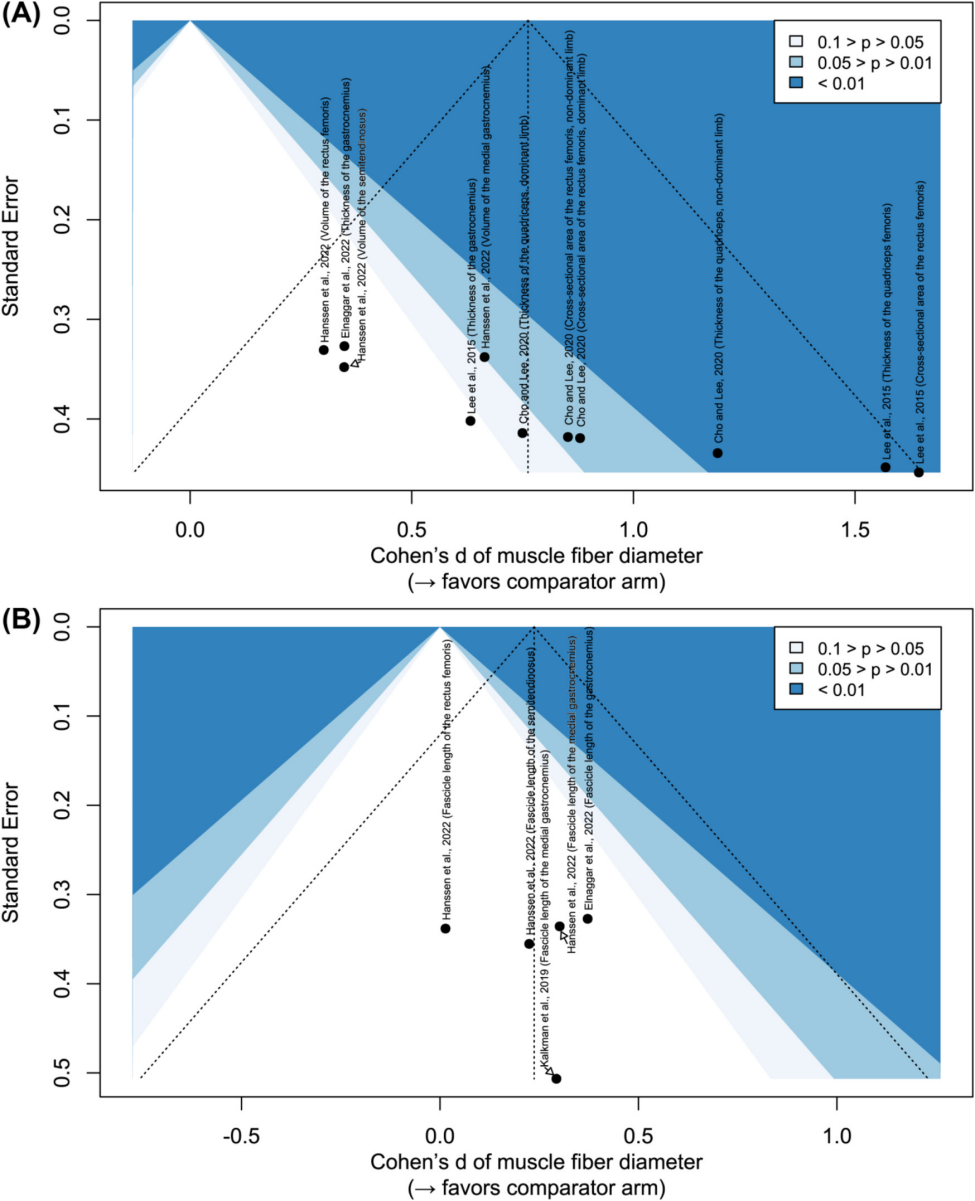
Contour-enhanced funnel plots: **(A)** effect on muscle fiber diameter; **(B)** effect on muscle fascicle length.

Figure 5 illustrates the risk of bias from potential missing publications. For muscle fiber diameter, results span all significance levels, suggesting no obvious publication bias. In contrast, only studies reporting non-significant improvements in muscle fascicle length have been published. Although the evidence is limited, these findings suggest that without innovative approaches, short-term (i.e., less than half a year) resistance or power training may have no effect on muscle fascicle length.

Three reviewed studies not included in the meta-analysis are presented for additional insights. In Kruse et al. (23), resistance training significantly improved vastus lateralis thickness but did not affect medial gastrocnemius thickness or fascicle length, while power training showed no differences in these measures. In Moreau et al. (25), resistance training produced a greater increase in the cross-sectional area of the rectus femoris than power training (*d* = 0.70, 95% CI: −0.32 to 1.72, with power training as control), whereas power training significantly enhanced rectus femoris fascicle length compared to resistance training (*d* = 1.20, 95% CI: 0.13–2.27, with resistance training as control). In Stackhouse et al. (26), adding neuromuscular stimulation to resistance training further improved the cross-sectional area of the quadriceps (d = 1.00, 95% CI: −0.31 to 2.32, with resistance training as control).

## 4 Discussion

An earlier meta-analysis (6) found that traditional gait training improves gait speed but not strength training in children with CP. Two subsequent meta-analyses (8, 9) provided updated evidence on the positive effects of strength training on mobility. The new contribution of the present review is its focused analysis of strength training modalities aimed at optimizing muscle morphology in children with CP, offering updated insights for guiding clinical practice during their developmental years.

Despite our effort to synthesize all evidence on the effectiveness of strength training, only ambulatory children with CP who demonstrated adequate cognitive function were recruited in the reviewed studies. This presents a significant limitation to the generalizability of the findings to children with severe motor impairments. While the overall prevalence of CP remains relatively stable (1), the severity, classified by GMFCS levels, varies across regions. In Australia, individuals with severe motor impairments (GMFCS levels IV and V) account for 29.3% of affected persons (27), similar to the 22.4% reported in Sweden (28). In contrast, in developing countries such as India, the proportion rises to 55.3% (29). Notwithstanding early evidence that strength training improves functional mobility in children with GMFCS IV (30, 31), its effects on muscle morphology remain unstudied, leaving a critical research gap for non-ambulatory children. This discrepancy underscores that the current findings and interpretations largely apply to children capable of following exercise instructions.

Although evidence on power training is limited (20, 25), current data strongly favor resistance training for increasing muscle fiber diameter. This is expected, as muscle hypertrophy during strength training is driven by loading effect—higher loads are more effective than submaximal loads in untrained individuals (32). Moreau et al. (25) compared fixed-speed, maximal loading with high-velocity, high-repetition submaximal loading, and their findings support the meta-analysis results. Moreover, performing more repetitions at lower loads until volitional failure leads to greater discomfort and fatigue without additional strength benefits (33, 34), which is less desirable for untrained children. Thus, current evidence favors using resistance training to enhance muscle fiber diameter in children with CP.

Despite limited evidence of muscle hypertrophy from power training, it is important to note that power and strength contribute to distinct aspects of lower extremity neuromuscular function. For instance, dynamic steady-state balance is strongly correlated with maximal strength in children (35), whereas gait speed is closely linked to peak power in children with CP (36). From a developmental standpoint, muscle strength precedes power, and together they achieve a full spectrum of neuromuscular capacity. It may be logical to prioritize resistance training for muscle hypertrophy, then integrate dynamic power training to enhance functional capacity in children with CP; however, further evidence is needed to confirm this sequencing.

While current evidence does not show a clear effect of resistance or power training on muscle fascicle length (CIs crossing zero), it would be premature to conclude that strength training yields minimal adaptations. In fact, Moreau et al. (25)—not included in the meta-analysis—demonstrated a very large effect size for power training compared to resistance training. A key difference is that Moreau and colleagues employed concentric training at 80% of maximum force, whereas other studies (20–22) used lower loads. For healthy, trained individuals, increasing muscle fascicle length requires accentuated eccentric-load strength training (37, 38). Overall, the literature indicates that higher loads, preferably utilizing power training principles (either concentric or eccentric), are necessary to enhance muscle fascicle length. Notably, most studies employed lower loads with body weight or weighted vests, which, while effective for hypertrophy, may not sufficiently target fascicle length. Ideally, strength-training programs for children with CP should progressively incorporate higher loads — 70%–85% of 1 RM for resistance training and 60%–80% of 1 RM for power training (7)—and clinicians should always fine-tune these loads to each child's GMFCS level and individual needs.

We suggest three future research directions to complement muscle mass and strength development protocols. First, joint intervention approaches warrant mention. Muscle strength—and by extension, function—in children with CP is influenced by spasticity (39). While it remains debated whether underdeveloped lower limb muscles directly contribute to spasticity, early reduction of spasticity appears to facilitate improvements in motor function. To address muscle spasticity in CP, treatments such as Botulinum neurotoxin type A (BoNT-A), selective dorsal rhizotomy, and extracorporeal shockwave therapy are available (40). For example, BoNT-A reduces spasticity and rigidity, improving lower limb movement and muscle tone (41). In CP, BoNT-A injections inhibit acetylcholine release at neuromuscular junctions, block nerve-muscle signal conduction, reduce spasms, and enhance joint mobility (42). Williams et al. (43) combined BoNT-A injections with functional resistance training in children with CP, resulting in significant muscle hypertrophy and functional gains. Thus, physiotherapists may consider combining invasive treatments with non-invasive strength training to optimize muscle adaptations in children with CP. Second, we echo the recent calls by Modlesky and Matias for precision nutrition interventions (44). Nutritional supplements are effective in enhancing muscle growth and function in various populations (45), including CP (46, 47). Therefore, integrating strength training with nutritional supplementation warrants further investigation. Third, children with CP are often born into families with low socioeconomic status (48, 49). Given their socioeconomic limitations, long-term participation in center-based rehabilitation is often impractical for these families. Future research should explore telehealth approaches for strength training protocols, which would involve training parents as personal trainers and conducting periodic follow-ups to monitor progress.

Finally, this review does not address the impact of different strength training modalities on functional motor capacity. However, it is clear that muscle morphology underpins proper motor function, with muscle strength developing before power, which in turn precedes overall motor capacity. Therefore, the primary finding remains that resistance training targeting muscle hypertrophy should be prioritized, with complementary power training following initial adaptations.

In conclusion, this focused review highlights the distinct effects of resistance and power training on muscle adaptations in ambulatory children with CP. Practical barriers, such as participant recruitment and long-term trial compliance, have impeded data collection and precluded consensus on optimal strength-training protocols. Nonetheless, current evidence favors initial resistance training to induce muscle fiber hypertrophy. If strength training principles established in able-bodied populations apply here, loads should be progressively raised to submaximal intensities (∼80% of 1 RM) and paired with power training to further promote fascicle lengthening and mobility; these protocols should be tested in long-term trials (6–12 months). In the meantime, the efficacy of strength training—and its optimal protocols—for children with CP who have severe cognitive and mobility impairments (GMFCS IV and V) remains largely unexamined. Future research should prioritize this cohort and assess whether integrating strength training, invasive interventions, and tailored nutrition yields synergistic benefits.
